# Supplementary figures and images for: Beyond high hopes: A scoping review of the 2019–2021 scientific discourse on machine learning in medical imaging
Source: PLOS Digit Health. 2023 Jan 31;2(1):e0000189. doi: 10.1371/journal.pdig.0000189 (PMC9931290; doi:10.1371/journal.pdig.0000189)

S1 File: Search strategy (Web of Science)


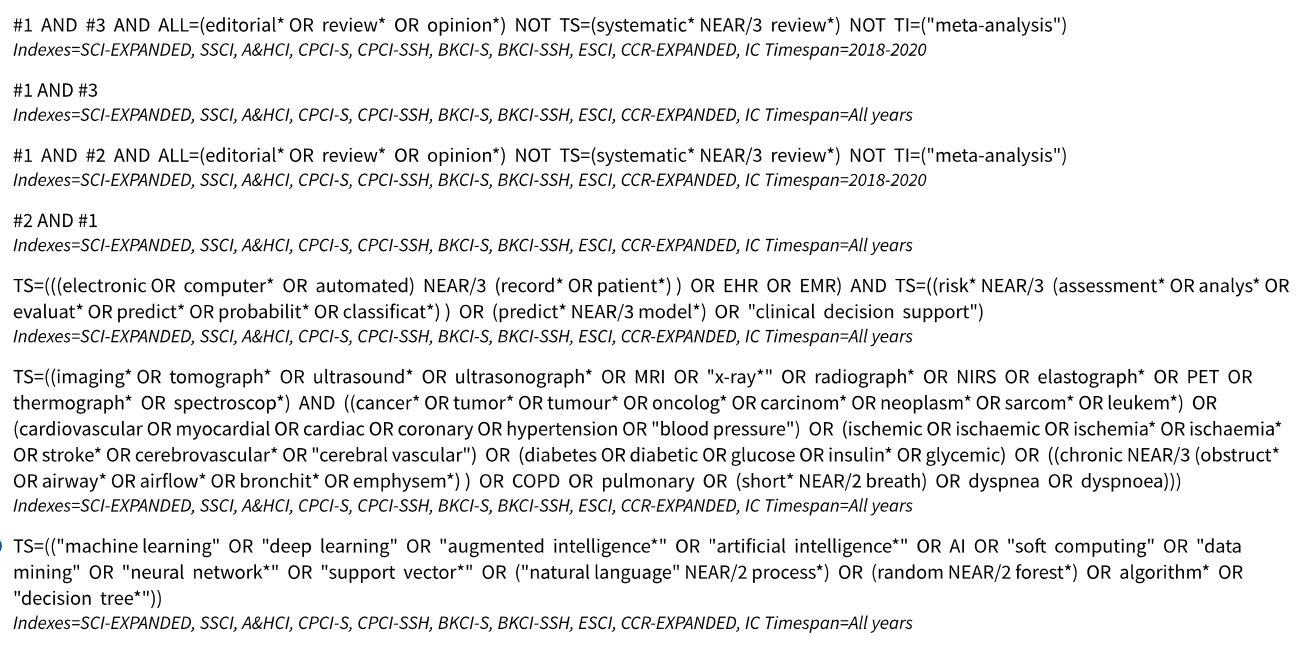

Supplement: S1 File — (DOCX) [file pdig.0000189.s001.docx]
