# Supplementary material for: Beyond high hopes: A scoping review of the 2019–2021 scientific discourse on machine learning in medical imaging
Source: PLOS Digit Health. 2023 Jan 31;2(1):e0000189. doi: 10.1371/journal.pdig.0000189 (PMC9931290; doi:10.1371/journal.pdig.0000189)
Supplement: Attachment — Submitted filename: Comments.docx [file pdig.0000189.s004.docx]

Thank you for this interesting and important topic, the well-written manuscript which greatly adds to the available literature. I have some minor concerns for the authors to consider:

1. In table 1 you included studies “Published after the 01.08.2019 and before 01.09.2021” for the first minute I was confused and thought that the first date was Jan 8th, I would request changing the date form.
2. Give percentages for your results, like:

Line 194 “Almost all publications (n=110)…..”

Line 204 “Most publications (n=102)…..”
