# Supplementary material for: Beyond high hopes: A scoping review of the 2019–2021 scientific discourse on machine learning in medical imaging
Source: PLOS Digit Health. 2023 Jan 31;2(1):e0000189. doi: 10.1371/journal.pdig.0000189 (PMC9931290; doi:10.1371/journal.pdig.0000189)
Supplement: Attachment — Submitted filename: Response_to_reviewers.docx [file pdig.0000189.s005.docx]

| Reviewer comments | Point -to-point replies |
| --- | --- |
| **Reviewer #1: Thank you for this interesting and important topic, the well-written manuscript which greatly adds to the available literature. I have some minor concerns for the authors to consider**:  1. In table 1 you included studies “Published after the 01.08.2019 and before 01.09.2021” for the first minute I was confused and thought that the first date was Jan 8th, I would request changing the date form.  2. Give percentages for your results, like: Line 194 “Almost all publications (n=110)…..” Line 204 “Most publications (n=102)…..” | Dear Reviewer. Many thanks for your positive comments & as well as remarks.  We have changed the date form accordingly.  We have adjusted accordingly to give percententages as suggested. |
| **Reviewer #2: The manuscript by Nittas et al is a scoping review of the scientific discourse around machine learning in clinical imaging. It identifies the promises and roadblocks that are put forward in published reviews of the field. I do not have much criticism to make. The manuscript is pleasant to read. With regards to the content, I trust the authors to have done a good job summarizing the selected articles. My biggest concern is that of sampling bias: how to ensure that the articles reflect well the points of view of the different scientific communities involved.**  Along this line, my most important concern is why limit to review with a focus on one of 5 chronic conditions? 56 reports were excluded because they did not focus on these conditions. I do not understand the logic. Rather, I see this restriction as a bias against broader reviews on the state of AI in health.  Another major concern that I have is that focus on the terms "AI" and "ML". Indeed, biostatisticians use the terms "prediction models" where others would use the term "ML". There is a gradient of techniques between good old statistical models (such as linear models) and more complex AI tools (such as deep learning). Linear models are still in many cases very relevant and can be seen as machine learning tools (they are studied by the machine learning research community). I note that 223 articles were excluded because of lack of focus on ML. Is this adding a bias against the vocabulary used by traditional biostatisticians, and thus downplaying the view of an important subcommunity? | Dear Reviewer. Many thanks for your positive comments & as well as constructive remarks.  We understand your concern and we also understand that this might be a question some readers might have. We therefore outline our rational in p.4 (lines 128-133)  We acknowledge that this might also pose a limitation and have therefore added it as a limitation in p. 21 (lines 633-640)  Thanks for pointing that out. We have actually use different terms, including predict* / probabiit* and classificat*.  Please see supporting file 1. We thus believe to have captured the vocabulary of traditional biostatisticians. |
| **Reviewer #3: The authors have provided a timely and needed scoping review of machine learning (ML) techniques in medical imaging. The strengths and promises of ML were well articulated in table 2 and the domain and subdomain separation of utility is well thought out.Unfortunately the biggest drawback for the manuscript in current form is that most of the discussion is general to ML and very few insights about ML applied to medical imaging.**  1.I did not get a clear sense of what imaging modalities are under discussion? The state of utilizing ML in modalities that are standard of care such as CT or ultrasound is different from say molecular imaging or magnetic resonance imaging? A section or sub-section dicussing what modalities are under the scope of this review will be very helpful.  2. The authors searched for five specific chronic conditions of cardiovascular disease , diabetes, stroke, chronic respiratory disease and cancer. But the authors do not comment on what differences did they find in these medical subfields. Are certain fields utilizing these techniques more ? Is it more useful to be thinking of ML in oncology where lesions fall under the “classification” problems than say in diabates where one if more dependent on blood analysis than imaging?  3. I also did not get a good sense of which ML algorithms are being used for in imaging data? Automated classification and regression are the main ML contributions.  Is the literature more skewed towards classification tasks? Or is ML being utilized for predictions?  4. All challenges listed in table 4 are applicable to all ML applications not just imaging applications? What is social bias in the context of medical imaging?    5.Future directions - Once again, the focus is on general ML vs. imaging specific literature. Some questions that maybe answered in this section are-  Do we have a sense of which imaging modalities are better suited to be married to physiological or genomic information?  Imaging ia more expensive but provides higher resolution insight than large amounts of physiological data from wearable sensors. What trade offs do the experts see in terms of utilizing ML on imaging vs physiological data?  5.The authors conclude -"Finally, the future of image-basedML is expected to shift towards multi-source, open access, and explainable tools, with mobile and wearables devices playing an increasingly important role”. Mobile and wearable devices are currently mostly providing physiological  data and not imaging data. The papers that the authors refer to (29, 38)  seem to refer to personalized  physiological data. Please clarify if  the review found any instances of personalized imaging being utilized for medical purposes.  I commend the authors for taking on a much needed scoping review.In its current form the review is more a scope of ML in general and most of those issues are well known to the ML community. It will be very helpful to refocus on specifics of ML in medical imaging. | Dear Reviewer. Many thanks for your constructive remarks. We have addressed them individually and believe that they helped us improve our manuscript considerably.  Thanks for pointing that out. We have added a paragraph on p. 7 (lines 244-248)  Thanks for this comment. We have revised accordingly and added sentences (examples) throughout the manuscript to highlight differences/main findings in each of these areas. Please see:  p.7, lines 250-255  throughout p. 10-11, lines 308-361  p.16, lines 508-511  p.16, lines 513-523  Thanks for pointing that out. We have added a description on included ML algorithms in the new paragraph on p. 7 (lines 248-255)  Thanks a lot for this remark. In order to be more imaging-specific, we have added a new table (table 3, p 9-10) with challenges that are primarily linked to images and imaging modalities.  We additionally added four new paragraphs explaining table 3 (p. 10-11, lines 308-361)  We also updated the solutions paragraph with some suggestions related to the challenges listed in table 3 (p 14, lines 426-431 & 443-445)  We additionally updated the discussion (p.17) to reflect these changes.  We have updated the future directions to reflect more image-related considerations. Please find the revised paragraphs in page 16.  DThanks for pointing that out. Here, we did not imply that mobile apps and wearables provide imaging data, but rather that the physiological data they provide may be combined with traditional imaging data to generate more comprehensive and powerful algorithms. We have adjusted the sentence to reflect that and avoid any misunderstandings (p.21, lines 692-693). We have also on p.16 (lines 508-511) regarding the use of multi-omic imaging.  Many thanks. Your remarks helped us improve the manuscript considerably & re-focus the paper’s scope to imaging. We believe in the added value of the revised manuscript. |
